# RINGdb: An integrated database for G protein-coupled receptors and regulators of G protein signaling

**DOI:** 10.1186/1471-2164-7-317

**Published:** 2006-12-16

**Authors:** Yu-Ching Fang, Wei-Hsin Sun, Li-Cheng Wu, Hsien-Da Huang, Hsueh-Fen Juan, Jorng-Tzong Horng

**Affiliations:** 1 Department of Life Science, National Central University, Chung-Li 320, Taiwan; 2 Department of Life Science & Institute of Molecular and Cellular Biology, National Taiwan University, Taipei 106, Taiwan; 3 Institute of Bioinformatics and Systems Biology, National Central University, Chung-Li 320, Taiwan; 4 Department of Biological Science and Technology, Institute of Bioinformatics, National Chiao-Tung University, Hsin-Chu 300, Taiwan; 5 Department of Computer Science and Information Engineering, National Central University, Chung-Li 320, Taiwan

## Abstract

**Background:**

Many marketed therapeutic agents have been developed to modulate the function of G protein-coupled receptors (GPCRs). The regulators of G-protein signaling (RGS proteins) are also being examined as potential drug targets. To facilitate clinical and pharmacological research, we have developed a novel integrated biological database called RINGdb to provide comprehensive and organized RGS protein and GPCR information.

**Results:**

RINGdb contains information on mutations, tissue distributions, protein-protein interactions, diseases/disorders and other features, which has been automatically collected from the Internet and manually extracted from the literature. In addition, RINGdb offers various user-friendly query functions to answer different questions about RGS proteins and GPCRs such as their possible contribution to disease processes, the putative direct or indirect relationship between RGS proteins and GPCRs. RINGdb also integrates organized database cross-references to allow users direct access to detailed information. The database is now available at .

**Conclusion:**

RINGdb is the only integrated database on the Internet to provide comprehensive RGS protein and GPCR information. This knowledgebase will be useful for clinical research, drug discovery and GPCR signaling pathway research.

## Background

G protein-coupled receptors (GPCRs), which represent the largest family of cell-surface receptors, mediate a variety of extracellular signals, modulating many intracellular responses [1]. A wide variety of GPCRs control the activity of enzymes, ion channels and transport of vesicles via the catalysis of the GDP-GTP exchange on heterotrimeric G proteins (Gα-βγ), the key players in transmembrane signaling [2,3]. Signal-induced conformational changes enhance the guanine-nucleotide-exchange activity of the receptor, leading to the release of GDP (and subsequent binding of GTP) by the Gα subunit [2]. On binding GTP, conformational changes of Gα allow the release of Gβγ and the subsequent engagement of effectors that are specific to each Gα subtype [2]. GPCRs are extremely important because 50% of all currently marketed drugs have action on specific GPCRs [4]. However, only 10% of GPCRs are targeted by these drugs, emphasizing the potential of the remaining 90% of the members of the GPCR superfamily for the treatment of human diseases [5]. Tissue-specific expression coupled with the availability of highly selective ligands determines the physiological roles of GPCRs [4]. Information regarding the localization of GPCR and signaling/regulatory molecules, signaling pathways, and the relationship between GPCR signaling and diseases/disorders facilitates the identification of drug targets and the development of drugs.

In addition to GPCRs and G-proteins, a family of signal modulators (the regulators of G protein signaling or RGS proteins) shows more restricted expression. These proteins are being examined as potential drug targets. RGS proteins are a large family of signaling proteins and share a conserved signature domain (RGS domain) that directly binds and activates G-alpha subunits, modulating G protein signaling. The major role of RGS proteins is to act as Gα GTPase-accelerating proteins (GAPs). They reduce GPCR signaling by accelerating the rate of GTP hydrolysis by the G-protein α-subunit, which leads to Gα-Gβγ reassociation [6]. Inhibiting the binding of the RGS-box to Gα•GTP in this case would lead to a prolonged lifetime of the Gα subunit in the GTP-bound state, enhancing the GPCR-stimulated response through increased levels of free Gα•GTP and Gβγ subunits [7]. Recent findings indicate that RGS proteins not only regulate G proteins but also bind to other signaling modulators [7]. At least 20 proteins (besides G-alpha subunits) have now been identified as direct binding partners for RGS proteins, and additional binding partners remain to be found [4]. Many RGS proteins that bind non-G protein signaling partners are expressed exclusively in specific brain regions [8,9], which agrees with the extensive diversity of neuronal and glial GPCRs and the signal modulation required for proper brain function [4], making these proteins attractive targets for possible therapeutic intervention. Evidence also indicates that RGS proteins are able to directly bind to GPCRs [4]. For example, the PDZ domain of RGS12 interacts specifically with the interleukin-8 receptor (CXCR2) [10]. RGS proteins may stabilize an active GPCR/G protein/channel complex to limit the diffusion time necessary for activation and deactivation [11,12]. Given the highly restricted expression of RGS proteins and the changes in levels of RGS proteins in response to various disease states [5], understanding the part that RGS proteins play as direct links between G proteins and other signaling pathways and their possible contribution to disease processes are important research goals [4]. However, little is known about the native tissue distribution and physiological functions of all RGS proteins and about the physiological significance of the interaction between RGS proteins and other signaling molecules [4]. Although current databases such as GPCRDB [13] and gpDB [14] provide information on GPCRs and/or G-proteins, there is not a comprehensive database that provides information on RGS, G-proteins, GPCRs, and the relationship between GPCR signaling and diseases/disorders.

To address this matter, we have developed an integrated biological database to provide complete and organized RGS/GPCR information. A knowledgebase that deposits the integrated RGS/GPCR data will be very useful for clinical research, drug discovery and GPCR signaling pathway research.

## Construction and content

### Data sources and contents

**Figure 1:**
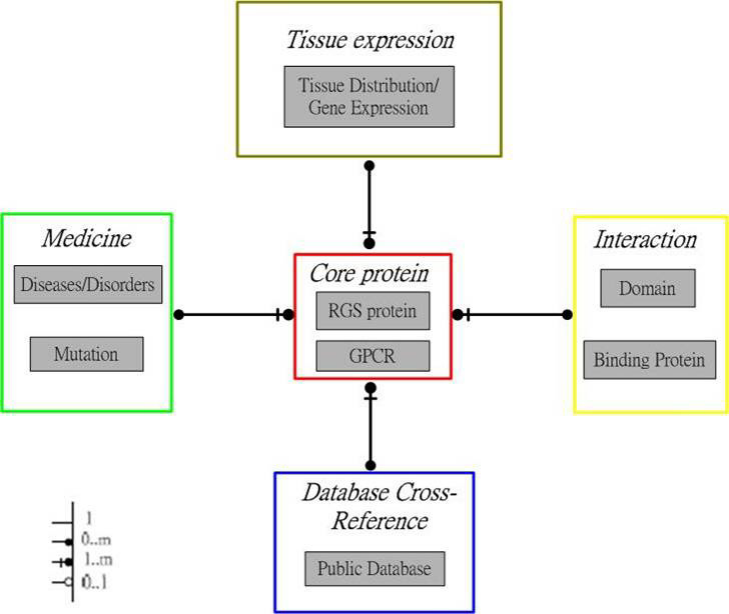
**The simplified relational scheme of RINGdb**. Each gray box represents an entity with various attributes. Most entities in our databases could be classified into five categories, *Core protein, Interaction, Medicine, Tissue expression *and *Database Cross-Reference*. For instance, each RGS protein may contribute to one or more Diseases/Disorders or not be discovered to relate to any Diseases/Disorders yet.

The RINGdb is a relational database implemented by MySQL on Linux operation system. We use the Apache web server and the PHP5 server side script engine. The web pages and all data parsers are written in PHP and Perl, respectively. Figure 1 shows the simplified relational scheme of our database. For example, each core protein, RGS protein or GPCR, in our database may interact with one or many proteins, have more than one known domains, distribute in various tissues, contribute to different diseases/disorders, have many artificial mutants or polymorphisms and could be referred to all kinds of public databases.

The RINGdb contains all types of information about mutations, tissue distributions, protein sequences, protein domains, post-translational modifications, protein-protein interactions, diseases/disorders, drugs and ligands. Most RGS protein information and GPCR diseases/disorders information is extracted from MEDLINE abstracts and full text papers (if available) by manual surveys. The informative sentences extracted from the literature are provided. The following lists are the major contents in RINGdb.

(i) The RGS protein *mutation *information is extracted from literature surveys. It describes what functional changes occur upon RGS mutations and provides mutation site information. GPCR mutation data have been primarily collected from GPCRDB [13]. GPCR mutation types, including mainly polymorphisms and laboratory artificial modifications, are also annotated but most RGS mutation data currently stored in RINGdb is generated by site-directed mutagenesis.

(ii) The *tissue distribution *data describes RGS/GPCR tissue specificity, distribution and expression. It is collected from the literature, Swiss-Prot [15], SOURCE [16] and GXD [17]. Tissue distribution information regarding RGS proteins was mainly retrieved from the literature. We also integrated Gene Expression Omnibus (GEO) [18] to our system as database cross-reference.

(iii) All RGS protein and GPCR *sequence *data is automatically retrieved from the Swiss-Prot database and only the protein sequences annotated by Swiss-Prot as standards are stored in RINGdb. At present, 2275 GPCR sequences and 109 RGS protein sequences are available in RINGdb. And most RGS proteins collected from Swiss-Prot belong to mammalian especially human, mouse and rat.

(iv) The protein *domain *data is collected from a literature survey, Pfam [19] and InterPro [20]. The literature derived domain information describes the potential RGS/non-RGS domain functions. The domain data extracted from Pfam and InterPro contains domain descriptions as well as their boundaries in RGS protiens or GPCRs. Most of the non-RGS domains of RGS proteins could confer additional functions regarding either the regulation of RGS proteins or the manner in which RGS proteins regulate effectors [5]. For inferring putative RGS binding proteins, we extract all putative domains interacting with the domains in RGS proteins from InterDom [21].

(v) The *post-translational modification (PTM) *data is extracted from the literature and Swiss-Prot. It contains information regarding modification sites, types and the description of the functional relationships between proteins and PTM.

(vi) The *protein-protein interaction *data about RGS proteins is obtained from literature surveys. It provides information on binding sites, binding partners and descriptions of the relationships between the protein and its binding partners. At present, 308 RGS-protein interaction entries are deposited in RINGdb. The size of protein-protein interaction data about RGS is much greater than that found in BIND [22], DIP [23] and MINT [24]. The numbers of RGS protein-protein interactions included in BIND, DIP and MINT are 38, 0 and 56. For inferring the relationship between GPCRs and RGS proteins, we also integrate GPCR and G-alpha protein interaction information from gpDB to RINGdb.

(vii) The *diseases/disorders *data is obtained through a search of the original literature. It contains information that describes the relationships between diseases/disorders and RGS proteins or GPCRs. Detailed disease information including disease definitions, syndromes and treatments is provided and the possible contribution of RGS proteins and GPCRs to certain disease processes is retrievable.

(viii) The *ligand *data automatically collected from KEGG [25] is available in RINGdb. It shows the names of GPCR ligands and the links to KEGG for examining detailed ligand information. We also provide the human, mouse and rat orphan receptor lists extracted from Swiss-Prot.

All the data deposited in RINGdb is focused on mammals including human, mouse and rat. For references available in PubMed, the links are provided. Furthermore, in the GPCR search page we provide links to GPCR classification methods, such as GPCRpred [26] and PRED-GPCR [27].

### Database cross-reference

For each RGS protein and GPCR, database cross-references are provided. The integration into the RINGdb system allows direct access to detailed information. All cross-references are classified into seven categories including Gene, Protein Domain, Medical, Protein-Protein Interaction, Pathway, Classification and Others. Basic introduction for the cross-referenced databases or resources is available when a mouse hovers over a link. This friendly interface allows RINGdb access and utility.

## Utility

### Query method

RINGdb provides an integrated, classified and user-friendly web interface for data query. Through the search and navigation page, users can perform several searches regarding particular RGS protein or GPCR entry by Protein Name/Keyword, Gene Name, Swiss-Prot ID and Swiss-Prot accession number. All queries are primarily classified into seven categories made up of: (i) General; (ii) Disease; (iii) Mutation; (iv) Tissue Distribution; (v) Protein-Protein Interaction; (vi) Protein Domain; (vii) Post-Translational Modification. In addition, users can navigate RGS proteins or GPCRs according to their protein families.

In order to retrieve disease information regarding the human RGS2 protein, for example, the user can specify 'Gene Name' search type, select 'Human' species, input RGS2 as the search term, choose 'Disease' information option and then press the send button. All diseases/disorders information involving the RGS2 protein can then be retrieved. The users may also select a particular disease/disorder such as Alzheimer's disease to find related RGS proteins or GPCRs. To find tissue distribution for human serotonin receptor 2B but not know its exact gene or protein name, as another example, the user is able to input the keyword 'serotonin' as search term, to choose 'Protein Name/Keyword' search type and to select the 'Tissue Distribution' information option. The returned page contains all GPCRs with the 'serotonin' keyword. The user then can click serotonin receptor 2B's gene name, HTR2B, for tissue distribution information. Not only does the user find tissue distribution information about RGS protein or GPCRs, but it is also possible to identify in which tissue both are co-expressed by 'Other Tissue Distribution information' search.

### Inference of the relationship between RGS proteins and GPCRs

**Figure 2:**
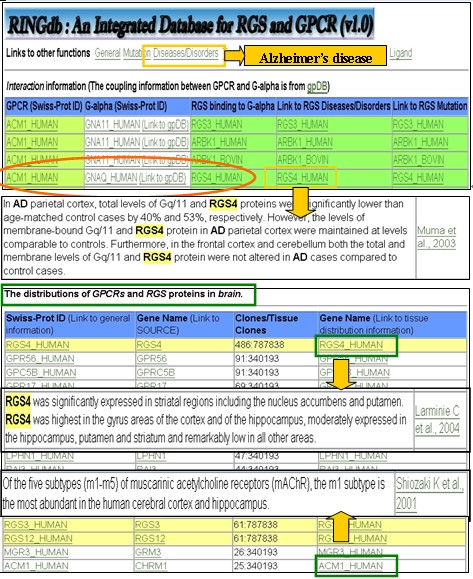
**Inferring relationships among GPCRs, Gα and RGS proteins for specific disease states**. The relationship among GPCR, Gα and RGS protein can be retrieved. The user can also find information regarding diseases that relate to such entries as well as information on their tissue distribution. Integrating the information presented above would be useful in inferring the relationship between RGS proteins and GPCRs.

In addition to comprehensive data query, one of most valuable aspects of the information available in RINGdb is the ability to infer the relationships between RGS proteins and GPCRs. If a RGS protein and GPCR both contribute to some diseases, participate in specific signaling pathways and have similar tissue distributions, they are likely to share a common functional role. Figure 2 shows the inference processes of the relationships among RGS proteins, GPCRs, Gα subunits and diseases/disorders. Users are able to search the interaction between the signaling molecules and find that both of RGS4 and CHRM1, the muscarinic cholinergic receptor M1 for the neurotransmitter acetylcholine, regulate the activity of the Gα q-linked pathway. In addition, according to the results of disease/disorder queries, users can find that both of them possibly related to Alzheimer's disease. Furthermore, they are both expressed in the hippocampus, a part of the vertebrate brain that is highly developed in mammals. The function of hippocampus appears to be related to the expression of responses that generate emotion and memory. Thus users may infer that RGS4 is a potential drug target for Alzheimer's disease. Finally, also using this database, users can carry out a comprehensive search on RGS4 including potential inhibitory sites and other binding partners, which might be needed for further studies. As the RINGdb contents from the literature increase, more integrated information can be retrieved simultaneously and helps users to discover the physiological functions of RGS proteins and GPCRs more accurately.

### Comparison between RINGdb and GPCRDB

The RINGdb differs from the current GPCRDB in many ways. Firstly, we have integrated the RGS proteins, a family of regulators of G-protein signaling, and GPCRs into RINGdb. Biologists interested in GPCR signaling can access RGS and GPCR information simultaneously and conveniently. Secondly, we provide an integrated, classified and friendly query interface. Users can access information faster, more simply and specifically. Thirdly, RINGdb focuses on clinical research related to GPCR signaling. The medical information about RGS proteins and GPCRs will have great usefulness in clinical research and drug discovery.

## Discussion

The analysis of several different properties of GPCRs and RGS simultaneously is useful in discovering new physiological functions that relate to disease. This information might prove important for the identification of novel drug targets and disease therapies. From the RINGdb search, we have found that the RGS domain of RGS1 may bind the cysteine-rich region of adenomatous polyposis coli protein (APC protein) which participates in Wnt signaling pathway. Both of RGS1 and APC co-express in germinal center B cells. It is likely that RGS1 is able to integrate G protein signaling and Wnt signaling through its interaction with APC. Furthermore, information from RINGdb has shown both of RGS1 protein and its known binding partners, including Gα i1, Gα i2, Gα i3, Gα o1 and α-2A adrenergic receptor, are present in multiple sclerosis (MS) lesions, suggesting they may have a role in pathological conditions. With information obtained from RINGdb, it is able to integrate useful known data to discover new putative signaling pathways in diseases. Although these putative signaling pathways require further experimental supports, it will indeed facilitate and direct research work.

## Conclusion

RINGdb is a novel integrated database providing RGS protein and GPCR information specifically for clinical research. Our study not only focuses on integrating all information related to RGS proteins and GPCRs into a biological database, but also helps to answer many different questions about the both proteins.

### Future works

Future prospects of this work include increasing database contents, in particular by adding information on three-dimensional protein structures, G-proteins and interactions between drug agents and GPCRs. Also, we intend to provide tools for visualization and analysis and to identify novel RGS and GPCR binding partners, thus enhancing the study of GPCR signaling pathways. Furthermore, we are planning to incorporate in the database high-precision data by mining and extract information from the literature in an automated way. Finally updating the system in real time is also our objective for future research.

## Availability and Requirements

RINGdb: an integrated database for RGS and GPCR (v1.0). 

This database is now available at .

## Competing interests

The author(s) declare that they have no competing interests.

## Authors' contributions

YCF carried out the database development, design, data collection, information extraction from the literature, programming and drafted the manuscript. YCF and WHS conceived of the database. WHS, LCW and HDH participated in the database design. WHS provided constructive suggestions from biologist viewpoint. WHS, HFJ, and JTH helped to draft the manuscript. All authors read and approved the final manuscript.
